# 4-[2-(Hydrogen phosphonato)-2-hydroxy-2-phosphonoethyl]pyridinium

**DOI:** 10.1107/S1600536811011408

**Published:** 2011-03-31

**Authors:** Feng-Lei Wang, Rong-Xin Yuan, Ji-Min Xie

**Affiliations:** a College of Chemistry & Chemical Engineering, Jiangsu University, Zhenjiang, 212013, Jiangsu, People’s Republic of China; b College of Chemistry & Materials Engineering, Jiangsu Laboratory of Advanced Functional Materials, Changshu Institute of Technology, Changshu, 215500, Jiangsu, People’s Republic of China

## Abstract

The title compound, C_7_H_11_NO_7_P_2_, exists as a zwitterion in which the positive charge resides on the protonated pyridyl N atom and the negative charge on one of the two phosphate groups. In the crystal, adjacent molcules are linked by O—H⋯O and N—H⋯O hydrogen bonds into a three-dimensional network.

## Related literature

For metal complexes of phospho­nic acids, see: Ma *et al.* (2008 ▶, 2009 ▶). 
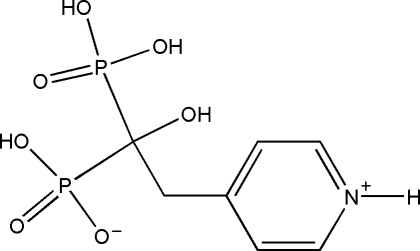


## Experimental

### 

#### Crystal data

C_7_H_11_NO_7_P_2_
*M* *_r_* = 283.11
Monoclinic, 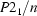

*a* = 10.083 (2) Å
*b* = 9.4713 (19) Å
*c* = 11.708 (2) Å
β = 94.87 (3)°
*V* = 1114.1 (4) Å^3^
*Z* = 4
Mo *K*α radiation
μ = 0.42 mm^−1^
*T* = 293 K
0.3 × 0.25 × 0.2 mm

#### Data collection

Rigaku Mercury diffractometer
Absorption correction: multi-scan (*CrystalClear*; Rigaku/MSC, 2005) ▶ *T*_min_ = 0.880, *T*_max_ = 0.92
11137 measured reflections
2548 independent reflections
2093 reflections with *I* > 2σ(*I*)
*R*_int_ = 0.051

#### Refinement

*R*[*F* ^2^ > 2σ(*F* ^2^)] = 0.051
*wR*(*F* ^2^) = 0.117
*S* = 1.02
2548 reflections
174 parameters
5 restraints
H atoms treated by a mixture of independent and constrained refinement
Δρ_max_ = 0.30 e Å^−3^
Δρ_min_ = −0.29 e Å^−3^

### 

Data collection: *CrystalClear* (Rigaku/MSC, 2005) ▶; cell refinement: *CrystalClear* ▶; data reduction: *CrystalClear* ▶; program(s) used to solve structure: *SHELXS97* (Sheldrick, 2008 ▶); program(s) used to refine structure: *SHELXL97* (Sheldrick, 2008 ▶); molecular graphics: *SHELXTL* (Sheldrick, 2008 ▶); software used to prepare material for publication: *PLATON* (Spek, 2009) ▶.

## Supplementary Material

Crystal structure: contains datablocks I, global. DOI: 10.1107/S1600536811011408/ng5136sup1.cif

Structure factors: contains datablocks I. DOI: 10.1107/S1600536811011408/ng5136Isup2.hkl

Additional supplementary materials:  crystallographic information; 3D view; checkCIF report

## Figures and Tables

**Table 1:** Hydrogen-bond geometry (Å, °)

*D*—H⋯*A*	*D*—H	H⋯*A*	*D*⋯*A*	*D*—H⋯*A*
O1—H1*A*⋯O2^i^	0.81 (2)	2.06 (2)	2.816 (3)	156 (3)
O4—H4*A*⋯O7^i^	0.81 (2)	1.77 (2)	2.574 (3)	176 (4)
O5—H5*A*⋯O2^i^	0.82 (2)	1.66 (2)	2.477 (3)	175 (4)
N1—H2*A*⋯O7^ii^	0.82 (2)	1.93 (2)	2.741 (3)	171 (4)
O6—H6*A*⋯O3^iii^	0.81 (4)	1.67 (4)	2.476 (3)	175 (4)

Symmetry codes: (i) 
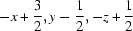
; (ii) 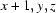; (iii) 
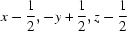
.

**Fig. 1.**
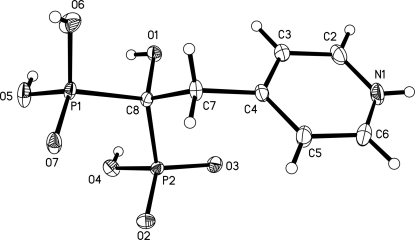
A view of the compound with the atomic numbering scheme. Displacement ellipsoids were drawn at the 30% probability level. [Symmetry code A = 1-x, y, 1/2-z].

**Fig. 2.**
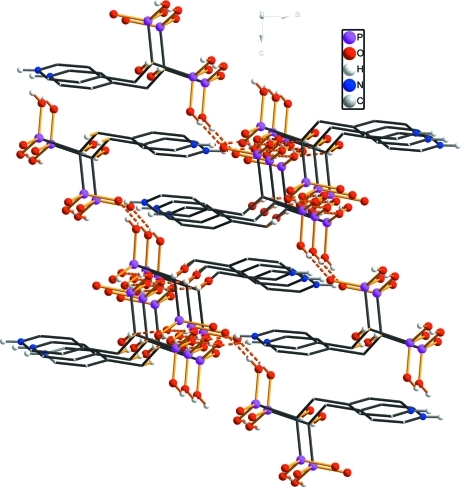
Hydrogen bonds connected three-dimensional structure of the title compound (Carbon-bond H atoms were omitted for clarity).

## supplementary crystallographic information

### Comment

Metal diphosphonates have been extensively studied for the diversity structures. Monomeric, dimeric, polymeric, two-dimensional layers to three-dimensional frameworks are all featured among these complexs Ma *et al.*, 2008; Ma *et al.*, 2009). Because diphosphonates have capabilities to stabilize transition metals in a wide range of oxidation states. The title compound was synthesized and characterized by X-ray crystal structure analysis.

There are some intermolecular hydrogen bonds in the structure of the title compound (Fig. 1 and Table 1). The intermolecular hydrogen bonds [O1—H1A···O2^i^, O5—H5A···O2^i^ and O4—H4A···O7^i^; symmetry code: -x+3/2, y-1/2, -z+1/2] bridge the molecules through head-to-tail into a one-dmensional chain. These chains are linked to two-dimensional structure through hydrogen bonds [N1—H2A···O7^ii^; symmetry code: x+1, y, z]. The hydrogen bonds [O6—H6A···O3^iii^; symmetry code: x-1/2, -y+1/2, z-1/2] connected the layers into a three-dimensional network (shown in Fig. 2).

### Experimental

The title compound was synthesized by reaction of 4-pyridine acetic acid hydrochloride (0.520 g, 3 mmol) and phosphite (0.711 g, 8.7 mmol) in chlorobenzene (15 mL). The solution was stirred while phosphorus trichloride (0.64 g, 4.7 mmol) was added drop by drop and the temperature should control between 110-120 ^o^C with vigorous stirring for 4 hours. The crude product was concentrated in vacuo. White block crystals formed in high yield by recrystallization.

### Refinement

Carbon-bond H atoms were positioned geometrically (C—H = 0.93 Å), and were included in the refinement in the riding mode approximation, with *U*_iso_(H) = 1.2*U*_eq_(C). H atoms bound to O and N atoms were located in a difference Fourier map and refined with restraints [N—H and O—H = 0.82 (1) Å, with *U*_iso_(H) values fixed at 1.5*U*_eq_(N) and 1.5*U*_eq_(O)].

### Crystal data

**Table:**

C_7_H_11_NO_7_P_2_	*F*(000) = 584
*M**_r_* = 283.11	*D*_x_ = 1.688 Mg m^−^^3^
Monoclinic, *P*2_1_/*n*	Mo *K*α radiation, λ = 0.71073 Å
Hall symbol: -P 2yn	Cell parameters from 10067 reflections
*a* = 10.083 (2) Å	θ = 3.4–27.6°
*b* = 9.4713 (19) Å	µ = 0.42 mm^−^^1^
*c* = 11.708 (2) Å	*T* = 293 K
β = 94.87 (3)°	Block, colorless
*V* = 1114.1 (4) Å^3^	0.3 × 0.25 × 0.2 mm
*Z* = 4	

### Data collection

**Table:**

Rigaku Mercury diffractometer	2548 independent reflections
Radiation source: fine-focus sealed tube	2093 reflections with *I* > 2σ(*I*)
graphite	*R*_int_ = 0.051
Detector resolution: 13.6612 pixels mm^-1^	θ_max_ = 27.5°, θ_min_ = 3.3°
dtfind.ref scans	*h* = −13→13
Absorption correction: multi-scan (*CrystalClear*; Rigaku/MSC, 2005)	*k* = −12→12
*T*_min_ = 0.880, *T*_max_ = 0.92	*l* = −15→15
11137 measured reflections	

### Refinement

**Table:**

Refinement on *F*^2^	Primary atom site location: structure-invariant direct methods
Least-squares matrix: full	Secondary atom site location: difference Fourier map
*R*[*F*^2^ > 2σ(*F*^2^)] = 0.051	Hydrogen site location: inferred from neighbouring sites
*wR*(*F*^2^) = 0.117	H atoms treated by a mixture of independent and constrained refinement
*S* = 1.02	*w* = 1/[σ^2^(*F*_o_^2^) + (0.0537*P*)^2^ + 1.150*P*] where *P* = (*F*_o_^2^ + 2*F*_c_^2^)/3
2548 reflections	(Δ/σ)_max_ < 0.001
174 parameters	Δρ_max_ = 0.30 e Å^−^^3^
5 restraints	Δρ_min_ = −0.29 e Å^−^^3^

### Special details

**Table:**

Geometry. All esds (except the esd in the dihedral angle between two l.s. planes) are estimated using the full covariance matrix. The cell esds are taken into account individually in the estimation of esds in distances, angles and torsion angles; correlations between esds in cell parameters are only used when they are defined by crystal symmetry. An approximate (isotropic) treatment of cell esds is used for estimating esds involving l.s. planes.
Refinement. Refinement of F^2^ against ALL reflections. The weighted R-factor wR and goodness of fit S are based on F^2^, conventional R-factors R are based on F, with F set to zero for negative F^2^. The threshold expression of F^2^ > 2sigma(F^2^) is used only for calculating R-factors(gt) etc. and is not relevant to the choice of reflections for refinement. R-factors based on F^2^ are statistically about twice as large as those based on F, and R- factors based on ALL data will be even larger.

### Fractional atomic coordinates and isotropic or equivalent isotropic displacement parameters (Å^2^)

**Table:**

	*x*	*y*	*z*	*U*_iso_*/*U*_eq_	
P1	0.66199 (6)	0.19546 (7)	0.09909 (6)	0.01961 (18)	
P2	0.87076 (6)	0.20407 (7)	0.30954 (6)	0.01825 (18)	
O1	0.88791 (18)	0.0416 (2)	0.12393 (16)	0.0229 (4)	
H1A	0.846 (3)	−0.021 (3)	0.151 (3)	0.041 (10)*	
O2	0.81307 (19)	0.3435 (2)	0.34177 (16)	0.0277 (4)	
O3	1.01876 (18)	0.1862 (2)	0.33689 (16)	0.0275 (5)	
O4	0.79155 (19)	0.0847 (2)	0.36597 (16)	0.0258 (4)	
H4A	0.827 (4)	0.008 (3)	0.370 (3)	0.059 (12)*	
O5	0.58172 (19)	0.0799 (2)	0.1556 (2)	0.0327 (5)	
H5A	0.613 (4)	0.000 (2)	0.157 (3)	0.069 (14)*	
O6	0.6632 (2)	0.1660 (2)	−0.02975 (18)	0.0341 (5)	
H6A	0.619 (4)	0.218 (4)	−0.072 (3)	0.059 (13)*	
O7	0.60449 (17)	0.33631 (19)	0.12698 (17)	0.0269 (5)	
N1	1.3345 (2)	0.3264 (3)	0.1400 (2)	0.0362 (6)	
H2A	1.4152 (19)	0.334 (4)	0.143 (3)	0.058 (12)*	
C2	1.2820 (3)	0.2028 (3)	0.1039 (3)	0.0331 (7)	
H2	1.3374	0.1275	0.0894	0.040*	
C3	1.1465 (3)	0.1875 (3)	0.0883 (2)	0.0279 (6)	
H3	1.1097	0.1020	0.0625	0.033*	
C4	1.0641 (3)	0.2998 (3)	0.1109 (2)	0.0221 (6)	
C5	1.1232 (3)	0.4260 (3)	0.1492 (3)	0.0334 (7)	
H5	1.0705	0.5024	0.1662	0.040*	
C6	1.2600 (3)	0.4372 (4)	0.1618 (3)	0.0413 (8)	
H6	1.2999	0.5220	0.1855	0.050*	
C7	0.9147 (2)	0.2907 (3)	0.0868 (2)	0.0234 (6)	
H7A	0.8954	0.2725	0.0055	0.028*	
H7B	0.8777	0.3826	0.1023	0.028*	
C8	0.8396 (2)	0.1788 (2)	0.1534 (2)	0.0166 (5)	

### Atomic displacement parameters (Å^2^)

**Table:**

	*U*^11^	*U*^22^	*U*^33^	*U*^12^	*U*^13^	*U*^23^
P1	0.0118 (3)	0.0169 (3)	0.0293 (4)	0.0002 (3)	−0.0028 (2)	−0.0001 (3)
P2	0.0170 (3)	0.0162 (3)	0.0213 (3)	0.0004 (3)	−0.0001 (2)	−0.0024 (3)
O1	0.0193 (9)	0.0172 (10)	0.0326 (10)	0.0020 (8)	0.0050 (8)	−0.0043 (8)
O2	0.0302 (10)	0.0183 (10)	0.0349 (11)	0.0020 (8)	0.0048 (8)	−0.0068 (8)
O3	0.0193 (10)	0.0355 (11)	0.0265 (10)	0.0036 (8)	−0.0060 (7)	−0.0050 (9)
O4	0.0287 (11)	0.0186 (10)	0.0310 (10)	0.0019 (8)	0.0082 (8)	0.0040 (8)
O5	0.0167 (10)	0.0234 (12)	0.0577 (14)	−0.0027 (8)	0.0011 (9)	0.0076 (10)
O6	0.0316 (11)	0.0390 (13)	0.0294 (11)	0.0093 (10)	−0.0116 (9)	−0.0025 (10)
O7	0.0161 (9)	0.0177 (10)	0.0466 (12)	0.0012 (8)	0.0018 (8)	−0.0016 (8)
N1	0.0154 (12)	0.0549 (18)	0.0386 (15)	−0.0072 (12)	0.0046 (10)	−0.0016 (13)
C2	0.0199 (14)	0.0435 (19)	0.0368 (16)	0.0047 (13)	0.0074 (12)	0.0006 (14)
C3	0.0225 (14)	0.0300 (16)	0.0319 (15)	−0.0022 (12)	0.0060 (11)	−0.0039 (12)
C4	0.0180 (13)	0.0260 (14)	0.0229 (13)	−0.0027 (11)	0.0051 (10)	0.0022 (11)
C5	0.0240 (15)	0.0265 (16)	0.0507 (19)	−0.0058 (12)	0.0094 (13)	−0.0057 (14)
C6	0.0262 (16)	0.043 (2)	0.055 (2)	−0.0144 (15)	0.0074 (14)	−0.0112 (16)
C7	0.0144 (12)	0.0248 (14)	0.0311 (14)	−0.0015 (11)	0.0021 (10)	0.0059 (12)
C8	0.0129 (11)	0.0129 (12)	0.0236 (12)	0.0017 (9)	−0.0004 (9)	−0.0007 (10)

### Geometric parameters (Å, °)

**Table:**

P1—O7	1.5015 (19)	N1—C2	1.339 (4)
P1—O6	1.535 (2)	N1—H2A	0.815 (19)
P1—O5	1.543 (2)	C2—C3	1.371 (4)
P1—C8	1.855 (2)	C2—H2	0.9300
P2—O2	1.5038 (19)	C3—C4	1.388 (4)
P2—O3	1.5090 (19)	C3—H3	0.9300
P2—O4	1.563 (2)	C4—C5	1.392 (4)
P2—C8	1.844 (3)	C4—C7	1.512 (3)
O1—C8	1.440 (3)	C5—C6	1.379 (4)
O1—H1A	0.806 (18)	C5—H5	0.9300
O4—H4A	0.811 (18)	C6—H6	0.9300
O5—H5A	0.821 (19)	C7—C8	1.551 (3)
O6—H6A	0.81 (4)	C7—H7A	0.9700
N1—C6	1.328 (4)	C7—H7B	0.9700
			
O7—P1—O6	114.23 (12)	C2—C3—H3	120.0
O7—P1—O5	108.10 (12)	C4—C3—H3	120.0
O6—P1—O5	109.93 (13)	C3—C4—C5	118.2 (2)
O7—P1—C8	112.34 (11)	C3—C4—C7	121.6 (2)
O6—P1—C8	103.49 (12)	C5—C4—C7	120.1 (2)
O5—P1—C8	108.59 (11)	C6—C5—C4	119.8 (3)
O2—P2—O3	116.23 (11)	C6—C5—H5	120.1
O2—P2—O4	107.85 (11)	C4—C5—H5	120.1
O3—P2—O4	111.15 (11)	N1—C6—C5	119.7 (3)
O2—P2—C8	108.99 (11)	N1—C6—H6	120.2
O3—P2—C8	106.16 (11)	C5—C6—H6	120.2
O4—P2—C8	105.97 (11)	C4—C7—C8	117.8 (2)
C8—O1—H1A	112 (2)	C4—C7—H7A	107.9
P2—O4—H4A	116 (3)	C8—C7—H7A	107.9
P1—O5—H5A	117 (3)	C4—C7—H7B	107.9
P1—O6—H6A	117 (3)	C8—C7—H7B	107.9
C6—N1—C2	122.5 (3)	H7A—C7—H7B	107.2
C6—N1—H2A	120 (3)	O1—C8—C7	107.8 (2)
C2—N1—H2A	117 (3)	O1—C8—P2	108.77 (16)
N1—C2—C3	119.8 (3)	C7—C8—P2	111.14 (17)
N1—C2—H2	120.1	O1—C8—P1	109.33 (16)
C3—C2—H2	120.1	C7—C8—P1	105.54 (16)
C2—C3—C4	119.9 (3)	P2—C8—P1	114.04 (13)

### Hydrogen-bond geometry (Å, °)

**Table:**

*D*—H···*A*	*D*—H	H···*A*	*D*···*A*	*D*—H···*A*
O1—H1A···O2^i^	0.81 (2)	2.06 (2)	2.816 (3)	156 (3)
O4—H4A···O7^i^	0.81 (2)	1.77 (2)	2.574 (3)	176 (4)
O5—H5A···O2^i^	0.82 (2)	1.66 (2)	2.477 (3)	175 (4)
N1—H2A···O7^ii^	0.82 (2)	1.93 (2)	2.741 (3)	171 (4)
O6—H6A···O3^iii^	0.81 (4)	1.67 (4)	2.476 (3)	175 (4)

Symmetry codes: (i) −*x*+3/2, *y*−1/2, −*z*+1/2; (ii) *x*+1, *y*, *z*; (iii) *x*−1/2, −*y*+1/2, *z*−1/2.
